# Transsternal and Transpericardial Approach to Descending Thoracic Aorta via Median Sternotomy—An Option for Extensive Aortic Surgery

**DOI:** 10.1055/s-0042-1750413

**Published:** 2022-12-15

**Authors:** Kay-Hyun Park

**Affiliations:** 1 Department of Thoracic and Cardiovascular Surgery, Seoul National University Bundang Hospital, Seoul National University College of Medicine, Seongnam, Korea

**Keywords:** elephant trunk, frozen elephant trunk, chronic dissection, extensive aneurysm

## Abstract

We describe a technique for approaching the distal descending thoracic aorta via median sternotomy and posterior pericardiotomy, which enabled us to perform the extensive aortic repair. While this approach shared the lesser invasiveness of the frozen elephant trunk procedure with less confinement by anatomic features, the advantage was counterbalanced by the high incidence of spinal cord ischemia. This approach can be an option in highly selected patients who require extensive aortic repair but have factors prohibiting other conventional approaches.

## Introduction

For surgical procedures involving the descending thoracic aorta, the left thoracotomy provides excellent exposure and allows extensive procedures such as combined replacement of the aortic arch and ascending aorta. 1 However, in the patients who require concomitant procedures on the aortic root or cardiac valves, the exposure provided by the lateral thoracotomy is suboptimal or prohibitive for performing an expeditious surgery. In addition, some patients carry conditions that increase the technical difficulty or morbidity of the lateral thoracotomy (old age, poor pulmonary function, dense pleural adhesion due to previous thoracotomy or pleural infection, etc.). Although the frozen elephant trunk (FET) procedure makes a single-stage repair possible for addressing the descending aortic lesion concomitantly with the proximal aortic repair or cardiac procedures, this is not free from a few anatomical prerequisites such as the need of adequate distal landing zone and the absence of large distal entry tear or true lumen compression by thrombosed false lumen in case of chronic dissection. 2

**Fig. 1:**
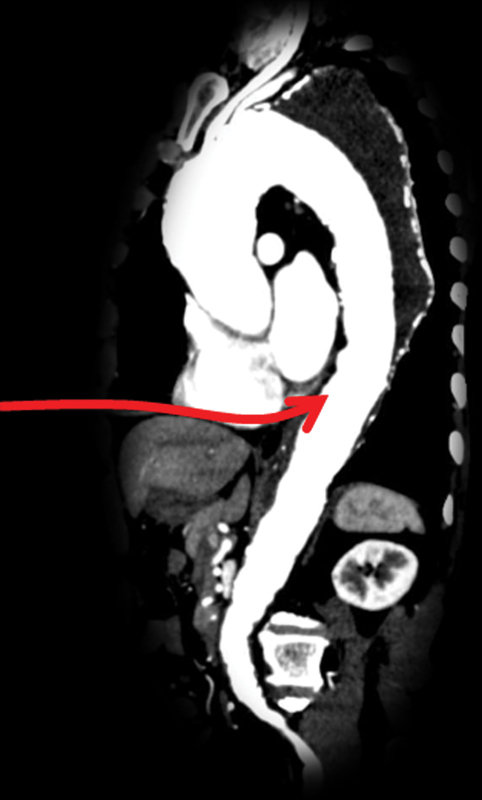
Sagittal section of computed tomography showing the proximity of the distal descending thoracic aorta to the heart.

We called attention to the fact that the distal descending thoracic aorta lies just behind the heart with the depth from the sternum not longer than that of the distal aortic arch ( Fig. 1 ). We have been using the median sternotomy for single-stage repair of various complex aortic pathologies for which a conventional strategy would have required two-stage repair or more extensive incisions. This article aims to describe the technical details and potential advantages and limitations of detouring the left thoracic cavity by approaching the distal descending thoracic aorta through a median sternotomy and posterior pericardiotomy.

From all patients whose medical information and computed tomographic (CT) scan images are presented in this article, informed consent was acquired for recording and publishing surgical videos and CT images for academic purposes. The novel nature of this technique was explained to the patients, along with the alternative options. Our institutional review board approved the acquisition and publication of patient data (IRB number B-2101-660-102).

## Patients

Patient details are given in Table 1 . For the sake of arrangement according to the etiology, the case number in the table is not in the chronological order of the operation date. Four patients (cases 1–4) had degenerative aneurysm involving extensive or multiple segments of the aorta. Case 5 had type I endoleak after thoracic endovascular repair (TEVAR) and a history of previous thoracotomy for chronic empyema. Two patients who had chronic Type B dissection (cases 6 and 7) had pre-existing arch aneurysm. There were five patients (cases 8–12) who had undergone emergency repair of acute Type I aortic dissection and developed false lumen dilatation starting from the previous anastomosis site at the upper ascending aorta. Case 13 had a history of two aortic operations (aortic valve repair including ring annuloplasty, and thoracoabdominal aorta replacement) and presented with severe aortic valve regurgitation and descending aortic graft infection causing recurrent septic embolism.

**Table 1:** Patient details

No	Age/sex	Previous operation	Diagnosis	Operation	TCA (min)	Operation/CPB (min)	ICU stay/admission (day)	Postoperative problem	Follow-up (months)
*Neo-DTA inside the native aorta:*									
1	62/M		Aneurysm	Ascending-DTA	70	295/155	1/13	Type II endoleak—TAAR (68 months)	69
2	66/F		Aneurysm	Bentall-DTA	96	395/269	4/66	Permanent paraparesis	24
3	75/F		Aneurysm	Ascending-DTA	75	265/148	27/112	Permanent paraplegia, tracheostomy—late death	18
4	82/F		Aneurysm	Ascending-DTA	81	260/159	3/19		26
5	62/F	TEVAR	Aneurysm	Ascending-DTA	77	335/165	1/15		20
6	59/M		Aneurine + chronic dissection	Ascending-DTA	55	300/153		Died of bowel infarction	0
7	69/M	Ascending	Aneurine + chronic dissection	David-DTA	66	450/271	13/124	Permanent paraparesis	10
8	43/M	Bentall	Chronic dissection	Ascending-DTA	98	445/266	5/27		59
9	68/F	Ascending	Chronic dissection	Bentall-DTA	94	415/230	4/15		61
10	67/M	Ascending	Chronic dissection	Ascending-DTA	94	369/213	2/18	Died of AEF	14
11	65/M	Ascending	Chronic dissection	Ascending-DTA	102	350/203	1/34	Temporary paraparesis, type II endoleak	55
12	75/F	Ascending	Chronic dissection	Ascending-DTA	72	355/179	4/28		9
*Extra-anatomic neo-arch/DTA* :									
13	52/M	Root repair, TAAR	Root aneurine + DTA graft infection	Bentall-arch + bypass to DTA	57	415/242	1/42		24
14	42/M		DTA stenosis + severe MR	MV repair + bypass to DTA	39	296/151	1/6		65
15	45/M		Root aneurine + coarctation of aorta	Bentall + bypass to DTA	22	282/167	3/23		48

Abbreviations: AEF, aortoesophageal fistula; AV, aortic valve; CPB, cardiopulmonary bypass; DTA, descending thoracic aorta; F, female; ICU, intensive care unit; M, male; MR, mitral regurgitation; MV, mitral valve; TAAR, thoracoabdominal aorta replacement; TCA, total circulatory arrest (arrest of descending aorta perfusion); TEVAR, thoracic endovascular aortic repair.

**Fig. 2:**
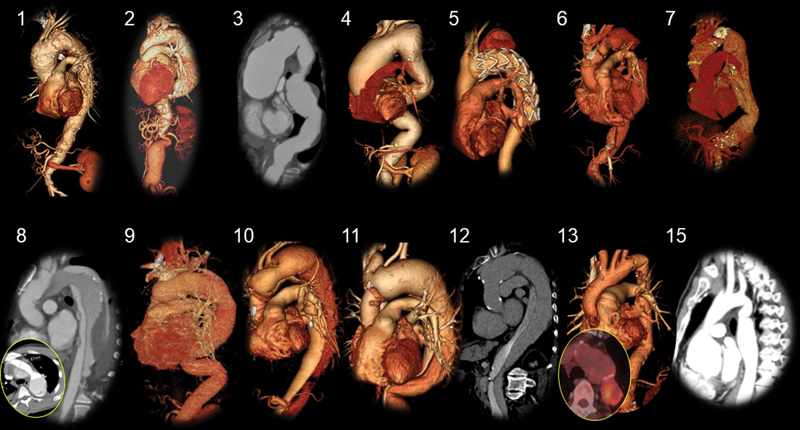
Preoperative computed tomography scan images of the patients. The numbers correspond to case numbers described in the text and Table 1 .

**Fig. 3:**
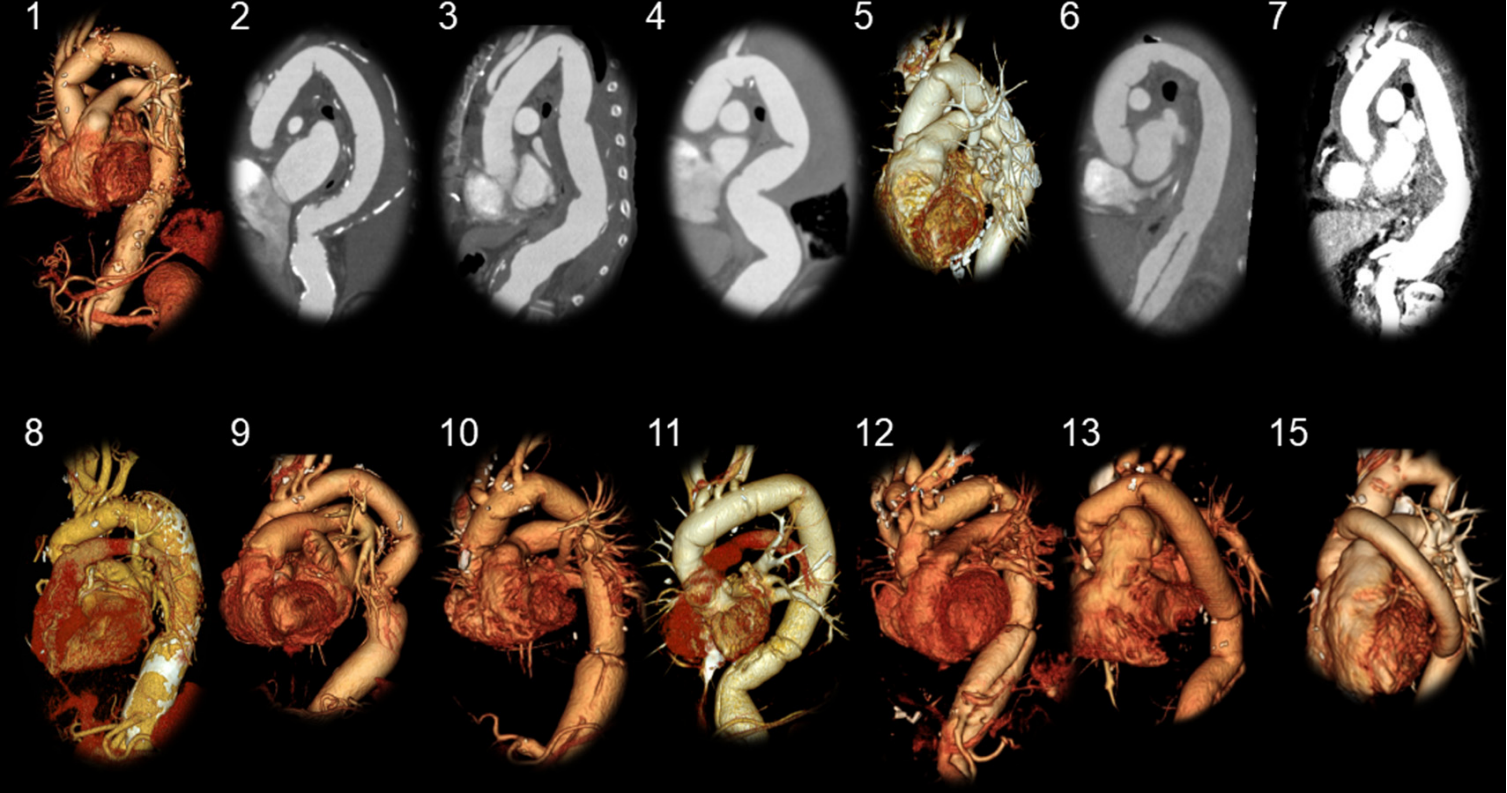
Postoperative computed tomography scan images taken within 14 days after the operation.

In the remaining two patients, the presenting pathology was severe stenosis of the descending thoracic aorta caused by aortitis of unknown etiology or congenital coarctation. Because the distal descending thoracic aorta had adequate size and quality on CT imaging, ascending-to-descending thoracic aorta bypass was performed concomitantly with the cardiac procedure (mitral valve repair and Bentall procedure). The preoperative and the early postoperative CT scan images taken during the hospitalization are shown in Figs. 2 and 3 .

## Surgical Techniques

### Access to the Descending Thoracic Aorta

Exposure of the descending thoracic aorta by incising the posterior pericardium is simple and straightforward. After cardiopulmonary bypass is commenced and the heart decompressed, the parietal pericardium behind the left ventricle is exposed by lifting the heart with the aid of an assistant's hand or by an apical suction device as used for off-pump coronary bypass grafting. The pericardium is incised longitudinally, and the descending aorta is identified and separated from the esophagus. The right-sided intercostal arteries and small mediastinal branches in the exposed field are ligated with metal clips. As this step takes only a few minutes while the patient is being cooled, this does not increase the operation time.

### In Situ Replacement



**Video 1** Transpericardial approach to the distal descending thoracic aorta for one-stage replacement of extensive aneurysm via median sternotomy (case 1).


**Fig. 4:**
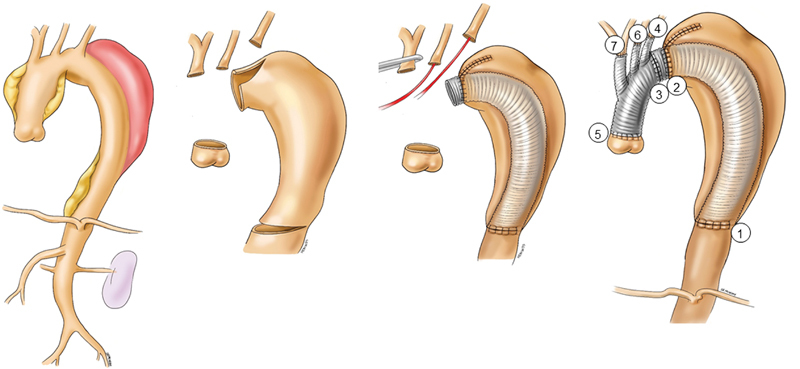
Replacement of almost entire thoracic aorta by a transsternal transpericardial approach to the descending thoracic aorta. The encircled numbers represent the sequence of anastomosis.

In 12 patients who underwent the single-stage repair of extensive thoracic aortic disease, the key elements of the technique have been the same as case 1, who was the first patient in our experience ( Video 1 ). Our usual approach to total arch replacement includes cannulation of the ascending aorta (or right axillary artery in case of “shaggy” aorta), cannulation of both venae cavae, deep hypothermia (nasopharyngeal 20 °C), and total circulatory arrest (TCA). The ascending aorta and the transverse arch are resected under total arrest without cerebral perfusion. When 20 minutes have passed since the start of TCA, selective antegrade perfusion of the right axillary, left common carotid, and left subclavian arteries is started (700 mL/min in total) after 2-minute-long retrograde cardioplegia (250 mL/min) for flushing of the brachiocephalic branches. The anterior half of the exposed descending aorta is incised transversely, and then a Dacron graft is passed from the proximal side to the distal aortotomy. Tagging the end of the graft to a chest tube aids easy passage. The anastomosis performed first is the fixation of the distal end of the graft into the distal aortotomy with double rows of continuous suture which bite the full thickness of the aortic wall and close the aortotomy at the same time. After closing the posterior pericardium, the proximal end of the intraluminal graft is folded inward and anastomosed to the arch stump with a continuous suture. Trimming and closure of the superior wall of the aneurysmal arch are needed to solve any size mismatch between the graft and large aortic opening. Then a separate four-branch graft is anastomosed to the proximal end of the intraluminal graft, which had been in-folded. After the left subclavian artery is reimplanted into a branch graft, systemic perfusion is resumed via a side branch graft. The remaining anastomoses are performed, while the patient is rewarmed. The proximal anastomosis to the sinotubular junction precedes reimplantation of the innominate and left common carotid arteries. By this point, the patient has been fully rewarmed. The heart resumes active beating, and the cardiopulmonary bypass is terminated ( Fig. 4 ).

**Fig. 5:**
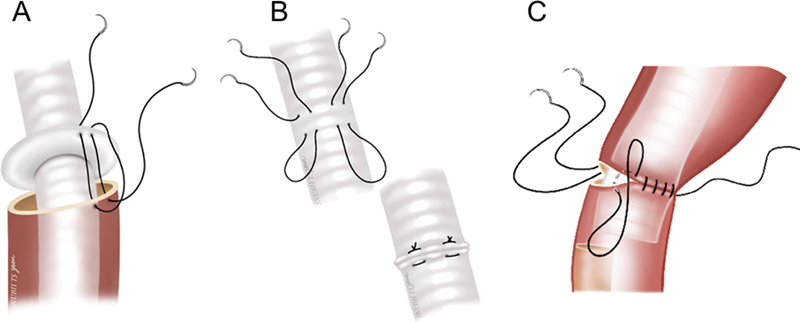
Methods of anastomosis—( **A** ) use of a graft with collar or ( **B** ) offhand suture cuff for the arch anastomosis and ( **C** ) inclusion technique with two rows of continuous suture leaving a short intraluminal trunk for the distal anastomosis.

A few modifications have been adopted in recent cases. First, the graft-arch size mismatch is managed by the use of a graft with a collar instead of the stepwise double anastomoses and trimming of the arch wall ( Fig. 5A ). Otherwise, a transverse ridge is made to be used as an offhand suture cuff, which we found helpful for expeditious suture work ( Fig. 5B ). Second, for the distal anastomosis to a large (>35–40 mm) descending thoracic aorta, a 5-cm-long short elephant trunk is left distal to the anastomosis in preparation for any possible future more distal operation ( Fig. 5C ).

When a concomitant Bentall procedure or valve-sparing root replacement is needed, this is done after completion of all of the other anastomoses. Intermittent retrograde blood cardioplegia is used for myocardial protection. Antegrade cardioplegic delivery via the coronary ostia is rarely used. The duration of circulatory arrest (lower body ischemia) and cardiopulmonary bypass time are approximately 20 minutes longer than those for our usual total arch replacement cases. However, the total operation time is not substantially prolonged and can be kept below 5 hours unless the operation is a redo procedure or requires a root replacement.

### Extra-anatomic Bypass



**Video 2** Bentall + total arch replacement with extra-anatomic (intrapericardial) bypass to the descending thoracic graft (case 13).


In three patients (cases 13–15), the Dacron graft was placed in the pericardial cavity along the lateral surface of the left ventricle. For case 13, who had infection with intraluminal vegetation in the proximal part of the descending aortic graft, the infected graft was excised as much as possible from both the proximal side (after excision of the arch) and the distal side (approached via the pericardium). Because the distal part of the descending thoracic graft showed minimal uptake in the 18F-fluorodeoxyglucose (FDG) positron emission tomography (PET) scan, it was saved and anastomosed in end-to-end fashion to a new graft. The other parts of the operation were not different from what was described above, except for the extra-anatomic, intrapericardial placement of the graft ( Video 2 ).

For the other two patients (case 14 and 15), although they did not require replacement of the arch, a short period of TCA was used for the end-to-side anastomosis between the graft and the descending thoracic aorta. The exposure was thought better than what would be provided by putting aortic clamps through the narrow pericardial incision. Deairing of the aorta was performed through the graft by slowly starting the aortic perfusion via the arch cannula that had been inserted for initiation of cardiopulmonary bypass. As the root or mitral valve procedure was done during the rewarming period, the use of TCA did not excessively prolong the operation.

## Potential Advantages

For the patients included in this series, one or more of the following options could have been chosen as the alternative approach: (1) two-stage repair using a conventional elephant trunk during the first stage, (2) one-stage repair via left thoracotomy or more extensive incisions such as bilateral thoracosternotomy (clamshell incision), and (3) proximal aorta replacement including the entire arch using a FET with or without later completion with endovascular repair.

The following limitations of those alternatives were taken into consideration in deciding that our patients were unsuitable or not ideal for them. Left thoracotomy is required for the first two options, and it may increase the risk of severe respiratory complications in elderly patients and those who have poor pulmonary function. In case the patient has dense pleural adhesion because of previous thoracotomy or pleural infection, technical difficulty and risk of pulmonary parenchymal damage are significantly increased. Concomitant replacement of the aortic root can be difficult, if not impossible, with the exposure provided by the left lateral thoracotomy. Although clamshell incision and T- or L-shaped sternothoracotomy can provide good exposure for the concomitant aortic root or cardiac procedures, these incisions share with the lateral thoracotomy the increased risk of respiratory complications.

Although the risks of morbidity and mortality of the individual procedures of two-stage repair may be lower than that of extensive one-stage repair, the rate of success in achieving the final treatment goal may not be higher because of the summated risk of two operations, danger of aneurysm rupture during the waiting period, and relinquishment of the second stage operation. 3 4 The use of FET is the most recently introduced and probably the most attractive among the alternative options because it can obviate the second-stage open surgery for the descending thoracic aorta in many cases. However, whether it is done as a definitive procedure or combined with later TEVAR extension, it is limited by the need of distal landing zone that is healthy and adequate in diameter. Especially in case of chronic dissection, the FET does not guarantee the successful exclusion of the enlarged false lumen if there is a large intimal tear below the stent-graft. 5 Although isolation of the thoracic false lumen from retrograde flow by the so-called false lumen procedure 6 has been introduced to promote the success, the outcome is yet to be proved by large-scale and long-term observations. 2 The development of distal stent-induced new entry is another unwelcomed problem of FET applied to chronic dissection. This has been reported to occur in as high as 33% and to inflict a negative impact on the prognosis. 7

Compared with the above options, the current technique shares with the FET procedure the advantage that single-stage repair is feasible by the less-invasive sternotomy, while being less restrained by the anatomical prerequisites needed for FET.

## Limitations

As shown in Table 1 , the postoperative recovery was smooth and did not require prolonged mechanical ventilation, prolonged intensive care unit stay, or subsequent hospitalization unless the patient developed the most dreadful complication, i.e., spinal cord ischemia (SCI). One patient died of massive bowel infarction which was caused by acute thrombotic occlusion of a superior mesenteric artery stent that had been inserted previously for alleviation of mesenteric malperfusion. Re-exploration for bleeding was required in one patient and no patient experienced stroke or significant renal dysfunction.

Despite the technical advantages, we think that the present technique should be considered only for highly selected patients because of the following problems we have experienced or been concerned about.

### Spinal Cord Ischemia

The high incidence of SCI makes us refrain from wider application or recommendation of the current technique. Excluding cases 14 and 15, 4 of 13 patients (30.8%) who underwent extensive aortic replacement developed SCI. Although three of the four patients partially or completely recovered the leg motor function, SCI was the major and only cause of complicated recovery and prolonged hospital stay.

Recently, we found that we were not the first that used the transpericardial approach to the descending thoracic aorta for extensive aortic replacement. Beaver and Martin reported a 14% incidence of permanent paraplegia in the article published in 2001. 8 The incidence of 14 to 30% is far from acceptable and greater than the incidence of SCI after the FET procedure, which has been reported to be 4.7% in a recent large-scale meta-analysis. 9 The incidence of SCI after exclusion of the aneurysm with the FET, whether it is frozen by metallic stents or surgical stitches, is significantly higher compared with the conventional one-stage extensive repair. The incidence of SCI is known to increase up to 12% when a longer (>15 cm) FET is inserted. 9 In contrast, Kouchoukos's group experienced only one case of SCI among 80 patients who underwent a clamshell incision approach to replace the aorta of comparable extent with our series. 10 Combined replacement of the arch and extensive thoracoabdominal aortic segments was not reported to increase the SCI incidence significantly. 1

Based on the aforementioned observations, it is to be emphasized again that the procedure described in this report should be considered only in the patients who are poor candidates for left thoracotomy. For low-risk patients, two-stage repair or other types of one-stage repair may be a safer option of extensive repair at least in terms of SCI. Of note is the technique introduced by Roselli et al, 11 which made it possible to detour the anastomotic constraints of chronic dissection and avoid second-stage thoracotomy. During total arch replacement via median sternotomy, they made a long fenestration in the intimal flap of the distal DTA by approaching it as we did, where the distal end of the stent-graft was landed during second-stage TEVAR. It ensured the complete exclusion of the thoracic false lumen with only 4% incidence of SCI. Such strategy could have been applied to some of our patients.

On reflection, some elements in our surgical strategy might have increased the risk of SCI. First of all, despite the longer TCA (arrest of descending aortic perfusion) duration (79.8 ± 15.8, 55–102 minutes) compared with conventional total arch replacement or FET procedure, we applied the same perfusion strategy. With the target of cooling set at the nasopharyngeal temperature of 20 °C, some patients' core truncal temperature was not lower than 28 °C, which may not be safe for the spinal cord under prolonged arrest. 12 The relatively low flow rate of antegrade cerebral perfusion and omitting left subclavian artery perfusion might result in hypoperfusion of the vertebrobasilar artery. The risk of spinal cord hypoperfusion could be aggravated because we did not insert a cerebrospinal fluid drain for this type of procedure. These considerations have been incorporated into our practice as experience accumulated: ensuring truncal temperature below 28 °C, selective perfusion of all brachiocephalic branches with higher flow, and resumption of cardiopulmonary bypass at an earlier stage after completion of the two anastomoses at the descending aorta.

We also pay more attention to the segmental steal of blood flow via the patent intercostal arteries excluded by the elephant trunk. Considering that the common factor in the surgically fixed elephant trunk and FET procedures is “exclusion” of the aneurysm instead of resection, this would be a plausible mechanism explaining the higher SCI incidence compared with the conventional procedures “replacing” the same extent of descending thoracic aorta. Based on this speculation, we obliterated all intercostal arteries visible in the exposed segment in the latter half of the series. Even with the technical changes mentioned above, the most recent patient developed delayed SCI after 8-hour-long hypotension (90–100 mm Hg systolic) during the first night, which suggests the importance of maintaining adequate perfusion pressure during the early postoperative period.

### Persistent Aneurysm

**Fig. 6:**
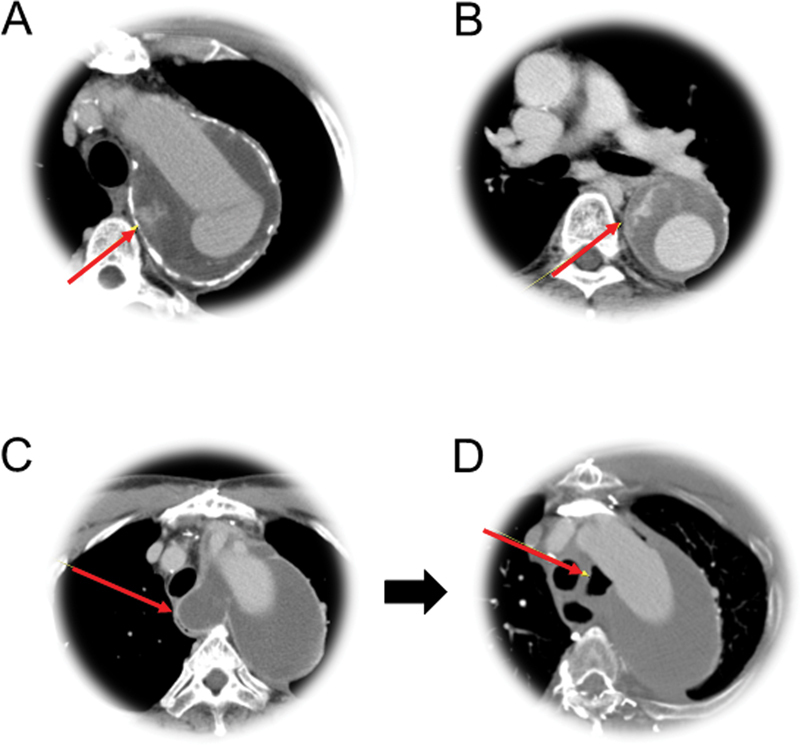
Late problems of the excluded aneurysm sac—( **A** and **B** ) retrograde filling from mediastinal branches and aortoesophageal fistula observed in case 1 and 10, respectively. ( **C** ) The thrombosed sac compressed the esophagus in the early postoperative image and eventually caused ( **D** ) perforation and mediastinitis 14 months later as evidenced by the air density around the graft.

In common with the FET procedure and TEVAR, persistent expansion of the excluded aneurysm sac was observed. In one of two such patients, the so-called type II endoleak from a mediastinal branch was demonstrated in the postoperative CT scan (case 1, Fig. 6A, B ). He underwent type II thoracoabdominal aorta replacement 69 months later. Type II endoleak was visible in another patient, and his aneurysm sac did not regress until the last CT examination done at 47 months after surgery. We think that such observations also justify the need for obliterating the branch vessels visible through the exposure.

### Aortoesophageal Fistula

Also in common with the aneurysm exclusion procedures such as FET and TEVAR, 13 14 our approach is not free from the risk of organ fistulation if a huge aneurysm sac does not shrink. Both aortoesophageal and aortopulmonary fistulas have been known to be a highly fatal long-term complication. The aneurysm of case 10 had been in contact with the esophageal wall in the preoperative CT scan. Complete thrombosis of the aneurysm sac without regression of the size led to erosion into the esophagus ( Fig. 6C, D ). The patient rejected surgical treatment and died shortly thereafter.

### Anastomotic Pseudoaneurysm or Rupture

Unlike the experience of Beaver and Martin, whose patients had a normal aortic diameter at the site of distal anastomosis, 8 most of our patients had dissection or aneurysmal dilatation extending beyond the diaphragmatic level. The average diameter of the aorta at the distal anastomosis was 36 mm with a range of 23 to 42 mm. This may lead to concern about size mismatch between the Dacron graft and the aorta and subsequent risk of pseudoaneurysm or rupture. During the midterm follow-up of our patients, no such problem was observed. Our anastomotic technique—two rows of sutures biting the full thickness of the aortic wall—is thought to be helpful to avoid the pitfall of the inclusion anastomosis, thereby minimizing the risk of pseudoaneurysm or the so-called Type Ib endoleak.

## Conclusion

Extensive repair of almost the entire thoracic aorta was feasible via sternotomy alone by approaching the descending thoracic aorta through the posterior pericardium and fixing a long elephant trunk with surgical stitches. This technique shares some advantages and complications of the FET procedure while being less confined by anatomical features. Balancing the advantage of less-invasive nature and the cost of high SCI risk, this technique can be considered in highly selected patients who require extensive aortic repair but have both physiologic factors prohibiting conventional extensive incisions and anatomic factors unsuitable for the FET procedure and subsequent endovascular completion.
